# Scaled Production of Functionally Gradient Thin Films Using Slot Die Coating on a Roll-to-Roll System

**DOI:** 10.1021/acsami.3c17558

**Published:** 2024-02-08

**Authors:** Tae-Joong Jeong, Xiaoqing Yu, Tequila A. L. Harris

**Affiliations:** Woodruff School of Mechanical Engineering, Georgia Institute of Technology, 813 Ferst Dr., Atlanta, Georgia 30349, United States

**Keywords:** coating, thin film, gradient structure, slot die coating, roll-to-roll, functional materials, functional gradients

## Abstract

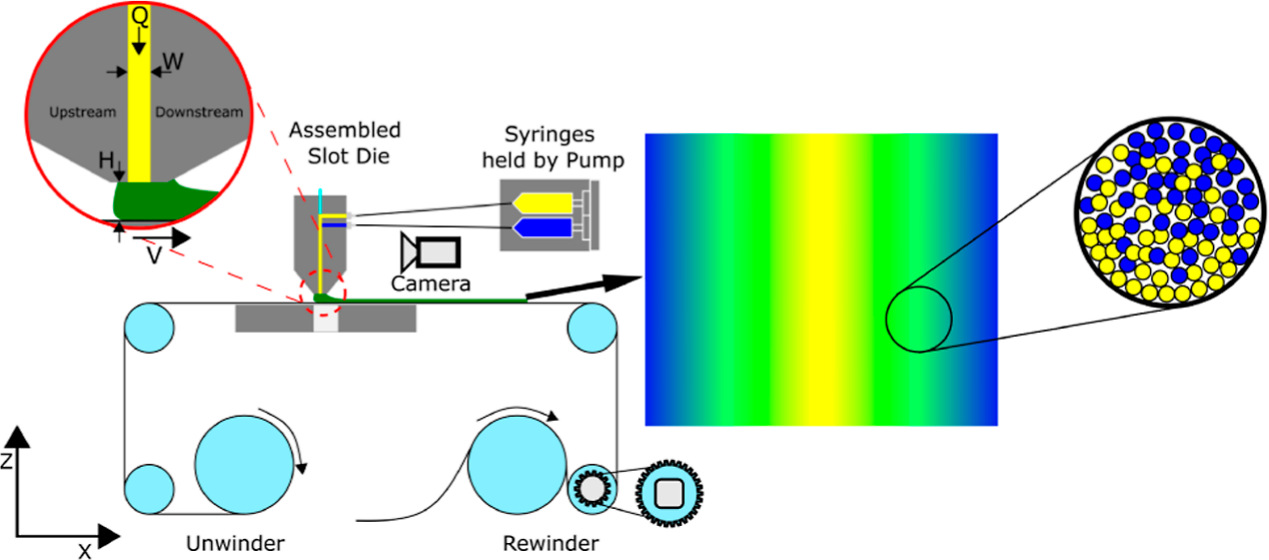

Polymer thin films with a cross-web gradient structure is a burgeoning area of research, having received more attention in the last two decades, for improvements in the performance and material properties. Such patterned films have been fabricated using several techniques, but in practice these techniques are non-scalable, material-dependent, wasteful, and not highly efficient. Slot die coating, a well-known scalable manufacturing process, is used to fabricate gradient polymer thin films which will be investigated herein. By incorporating slot die with the custom roll-to-roll imaging system, gradient thin films are successfully fabricated by forcing two fluidic materials into the slot die simultaneously and by manipulating the viscous, diffusive, and inertial forces. The materials will be allowed to intermix, with the aim of having approximately a 50% mix along the centerline of any two contiguous stripes. Moreover, several characterizations such as FTIR, UV–vis spectroscopy, and SEM are performed to assess the quality of the gradient polymer thin films. The gradient structure fabricated using functional and nonfunctional materials has successfully improved the functional properties compared to fully blended two materials. This work will provide an understanding of the mechanisms to obtain gradient polymer thin-film structures that exhibit the desired geometric structure and performance.

## 1 Introduction

Recently, gradient thin films have received more interest due to their versatility in a plethora of research fields such as, but not limited to, packaging of flexible electronics,^1−3^ controlled cell growth in lab-on-a-chip (biosensors),^4^ thin-film electrical devices^5^ and production of nanopaper.^6^ Gradient structures are advantageous because they have been shown to improve the properties of materials,^7^ such as electrical,^8,9^ thermal,^10^ adhesive,^11^ and mechanical^12^ properties. The enhancements of these properties can be realized by fabricating the gradient thin film such that the gradient interface is formed through the thickness^13−15^ or along the sidewalls^1−7^ of each coated material. These structures can be composed of two or more different materials^6,8,10,11^ or by using different concentrations of the same material.^1,5,12^ Gradient structures are utilized in large and small area applications such as secondary battery,^16,17^ flexible electronic,^18,19^ and origami film^20^ industries due to the efficiency of the method and the structure itself. More specifically, the gradient structure of an organic electrically conductive material, poly(3,4-ethylenedioxythiophene):polystyrenesulfonate (PEDOT:PSS), was developed in application areas such as wearable smart electronics^2^ and optoelectronics.^21^ Further, polyethylenimine (PEI) is a synthetic cationic material, and the gradient structure has been used in many applications such as nanofiltration membranes^22^ and cell adhesion.^23^ However, the gradient structures in these applications mostly exist through the thickness and/or use lab-scale methods that are not scalable and inefficient.

Coating techniques to achieve the desired gradient pattern and structure of these thin films from liquid solutions include spin coating,^1^ inkjet printing,^24^ spray coating,^6^ photolithography,^2,25^ filament writing,^3^ and cyclic draining-replenishing.^26^ Being primarily used at the lab scale, these techniques have critical limitations and challenges in terms of scalability, material selection, and waste production.^27−32^ Among many well-known coating methods, slot die coating^33^ is a premier technique for scaling thin films with predictable material properties and structures for various patterns such as wide regions,^34,35^ stacked bilayers,^36^ patch coating,^37,38^ and side-by-side alternating stripes.^39,40^

Gradient patterns were developed using both active and passive mixing mechanisms. For active mixing, an external force is applied to allow mixing to occur, which includes acoustic,^41^ thermal,^42,43^ and magnetic actuators.^44−46^ Even though active methods have been successful in inducing microfluidic mixing, they are very complex. Passive mixing in microfluidics occurs as multiple fluids simultaneously pass through a micro-sized flow cavity. Diffusion is one of the methods for passive mixing that requires a long time for mixing. However, microfluidic mixing allows for chaotic mixing to occur, in which advection aids diffusion for mixing. Moreover, the efficiency of mixing improves as the contact area of the fluids are larger, and the disturbance of fluid flows allows for an increase in passive mixing.^47^ Therefore, channel design has the highest impact on mixing as well as on certain parameters, such as the flow rate and viscosity. Cavity shapes like T-junction,^48^ Y-junction,^49^ and flow splitter^50,51^ have been successfully used as channel designs. However, the junctional geometries have worked best at very low flow rates (50–500 nL/min), which are not generally favorable for scaled manufacturing. Like the active mixing techniques, the flow-splitter geometries are complex; thus, new geometries that allow for significantly higher flow rates while mixing are needed.

Cubaud and Mason^52,53^ investigated a flow channel geometry that allows mixing to occur in a planar geometry for a wide range of fluid properties and flow parameters for microfluidic devices. Mixing was induced by controlling the flow dynamics of two fluids flowing through a planar geometry with periodic microchannels, based on the relationships between the Peclet number, viscosity ratio, and flow rate ratio of the two fluidic materials. While Cubaud and Mason^52^ demonstrated that fluid gradients can be formed, the work was limited to the internal geometry of a closed microfluidic device. Therefore, it is not understood whether such structural gradients can be maintained beyond the constrained and confined geometry of the microchannels to allow for gradient coatings.

In this work, we introduce the viability of scaling functionally gradient thin-film structures using slot die coating on a roll-to-roll (R2R) manufacturing system and geometries similar to those of Cubaud and Mason^52^ for the shim structure. Here, PEDOT:PSS and PEI are used as functional materials along with poly(vinyl alcohol) (PVA) as the secondary material to promote gradient structures that improve the electrical conductivity and adhesive properties, respectively, using a stable and scalable coating process for mixing and coating. Experimental analyses are used to verify the formation and stability of the gradient structure, as the fluids flow through the slot die and onto the substrate. Microstructural, adhesive, and electrical properties of functionally gradient thin films are analyzed to understand the quality and novelty of the gradient structured film.

## 2 Materials and Methods

### 2.1 Coating Fluids

PVA, specifically Mowiol 4-88 purchased from Sigma Alderich Corp., was used as a nonfunctional fluid phase. PVA was prepared by dissolving a specified mass of PVA in deionized (DI) water while stirring on a 60 °C hot plate with a magnetic stirrer for 30 min. PVA solutions of concentrations 7.5, 10, 15, and 20 wt % were made. The chemical structure of PVA is shown in Figure S1a. To qualitatively analyze the gradient coating, yellow food coloring (FD&C Yellow 5) was added to the center PVA fluid and blue food coloring (FD&C Blue 1 and Red 40) was added to the coating fluid flowing on each side of the PVA fluid stream. A drop of food coloring was added for every 2 mL of PVA. All the food dyes were purchased from McCormick Assorted Food Colors & Egg Dye. The properties of various concentrations of PVA are given in Table 1.

**Table 1:** Properties of PVA,^54^ PEDOT:PSS, and PEI^55^ Solutions at Room Temperature (20 °C) and Pressure (1 atm)

fluid	density (kg/m^3^)	viscosity (m kg/m s)
7.5 wt % PVA	1015	15
10 wt % PVA	1020	40
15 wt % PVA	1030	200
20 wt % PVA	1045	800
PEDOT:PSS	1000	35
25 wt % PEI	1080	400

Two functional materials were used, PEDOT:PSS and PEI. Clevios PH 1000 PEDOT:PSS was purchased from Heraeus and used as purchased. PEDOT:PSS served as an organic thermoelectric material. The properties of PEDOT:PSS are also given in Table 1. PEI was purchased from Fisher Scientific (∼M.N 60,000, 50 wt % aqueous solution, branched). 25 wt % PEI aqueous solution was made by dissolving 50 g of 50 wt % PEI in 50 g of DI water for 5 min at room temperature. PEI acts as a synthetic adhesive material. The chemical structures of PEDOT:PSS and PEI are shown in Figure S1b,c respectively.

Gradient thin films were formed using various combinations of solutions such as 20 wt % PVA/10 wt % PVA, PEDOT:PSS/7.5 wt % PVA, and 25 wt % PEI/10 wt % PVA, as shown in Table 1.

### 2.2 Slot Die Coating

The slot die coater was made of transparent poly(methyl methacrylate) (PMMA), conventionally known as acrylic glass, to allow for visual inspection of the internal flow. In this work, 0.1 mm thick polyethylene terephthalate (PET), purchased from Goodfellow Ltd. was used as the substrate and carrier web on the R2R manufacturing system.

A schematic of the R2R, used to coat the gradient thin film, is shown in Figure S2. The slot die was mounted such that a high-resolution camera could capture the internal flow and deposition of the coating fluid. The carrier web speed (*V*) and the flow rate of each fluid (center fluid *Q*_1_ and side fluid *Q*_2_) were controlled by a motorized roller and two syringe pumps, respectively. The coating gap, *H*, was set to create a small distance between the bottom of the slot die and the substrate. After coating, the gradient films were dried in a 60 °C oven for 30 min. The PEDOT:PSS/PVA gradient thin films were dried in a 60 °C oven for 1 h.

The shim, a sheet placed between the two slot die halves along the perimeter to create a desired offset distance *G* and the microchannel pattern that promotes mixing, was made of 0.25 mm thick PET, purchased from McMaster-Carr. The PET shim is designed following the work of Cubaud and Mason.^52^ As shown in Figure S3, the flow cavity consists of four microchannels with a minimum width of 0.2 mm, to induce more mixing. The outlet width was set as 1 cm to match the desired width of the gradient film. Three inlet ports were cut into the shim to allow for each fluid stream (center fluid and two side fluid streams) to be introduced independently.^52^

### 2.3 Wetting and Surface Property Measurements

Wetting and surface properties of PVA were analyzed to ensure good spreading of the coatings. The contact angle measurements were made to determine wettability using a Ramé–Hart goniometer (model, 500-U1) under ambient conditions following the ASTM 7334-08 standard. The contact angles of the coating fluids were measured on the substrate and the slot die materials, using the sessile drop method under ambient conditions.

### 2.4 Characterization of Gradient Films

The microstructure, geometry (width and thickness), and interfaces of the gradient thin film were analyzed using various microscopy techniques. A Phenom XL G2 scanning electron microscope was used to visually inspect the cross-section of the gradient thin film. A Nicolet iS 5 Fourier transform infrared (FTIR) spectrometer was used to analyze the chemical structure of the film, which can also be leveraged to verify the existence of a gradient structure. An Agilent Cary 60 UV–vis spectrophotometer was used to analyze the gradient color scheme and the level of mixing.

Adhesion tests were performed on samples to understand the durability of the materials. While not quantitative, the peel tests conducted were standard. The adhesive strength of the gradient thin film was measured using a cross-cut scratcher and Scotch tape following the ASTM D3359 standard. This method provides a qualitative understanding of the adhesivity.

Electrical conductivity was measured by using the four-point probe method. The probe contacts are lines of polished copper with an overall width of 4.6 mm, each probe having a width of 25.5 μm. The fabricated films were then cut to 4.6 mm width, and the measurements were made along the centerline, side, and gradient regions of the thin film.

## 3 Results and Discussion

### 3.1 Properties of Solutions

The contact angle and surface tension measurements of PVA, PEDOT:PSS, and PEI on PET and PMMA are provided in Table 2. DI water, which has a very poor wetting property and causes dewetting, has a contact angle of around 74°^56^ on the PET surface. From experimental experience, it was noticed that when the contact angle between the fluid and the solid is less than 60°, it is considered coatable. As shown in Table 2, the contact angle values of PVA are coatable but PEDOT:PSS and PEI do not meet the criteria. However, to coat these dewetting materials, different techniques are used. The details will be mentioned below.

**Table 2:** Measured Contact Angles and Surface Tension Values of Fluidic Materials

fluid	contact angle on PET (deg)	surface tension, σ (mN/m)
7.5 wt % PVA	55	44
10 wt % PVA	51	44
15 wt % PVA	52	44
20 wt % PVA	52	44
PEDOT:PSS	74	70
25 wt % PEI	69	64

### 3.2 Formation of Gradient Thin Films

Different combinations of PVA concentrations were used to fabricate gradient thin films via slot die coating. Based on the modified Peclet Number (Pe) formed by Cubaud and Mason,^52^ the flow rates through each inlet are calculated using eq 1.^52^
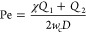
1

In the general form, Pe represents the ratio between the advective transport rate and the diffusive transport rate. In eq 1, χ is the viscosity ratio between the center fluid and the side fluid where the center fluid has a higher viscosity, which is necessary to promote mixing. As explained in the previous work, the fluid with the highest viscosity is maintained as the center fluid because the less viscous side fluid acts as the lubricant to the center fluid, which decreases the overall viscous effect. *Q*_1_ represents the mass flow rate at the center inlet, *Q*_2_ represents the mass flow rate through each side channel, *w*_c_ is the width at the channel outlet, and *D* represents the diffusion coefficient (*D*) of the solute material. The range of Pe that would induce internal mixing of two miscible fluids within the planar geometry is known to be between 5000 and 15,000.^43^

The *w*_c_ value for all experiments was set as 1 cm. The χ, *Q*_1_, and *Q*_2_ values for each combination material were altered to meet the Pe criteria as given in Table 3. Hence, a range of flow rates, *Q*_1_ and *Q*_2_, are needed for each fluid combination, and the viscosity ratio, χ, is set based on the materials of interest. Since χ is dependent upon the materials used, understanding the influence of χ is beyond the scope of this work. The values of *D* for the PVA solutions range from 1.26 × 10^–9^ to 2.00 × 10^–9^ depending on their concentration. The *D* for PEDOT:PSS is 1.5 × 10^–12^ m^2^/s,^57^ and for 25 wt % PEI it is 2 × 10^–10^ m^2^/s.^58^

**Table 3:** Flow Rate Calculation following Equation 1 for Each Combination of the PVA Concentration

index/sample #	center fluid	side fluid	χ	*Q*_1_ (mL/min)	*Q*_2_ (mL/min)
S1	20 wt % PVA	10 wt % PVA	20	0.6–1.5	0.3–1.0
S2	PEDOT:PSS	7.5 wt % PVA	2.33	0.9–1.1	1.1–1.3
S3	25 wt % PEI	10 wt % PVA	10	0.8–1.0	0.6–1.0

#### 3.2.1 Effect of the Flow Rate

**Figure 1:**
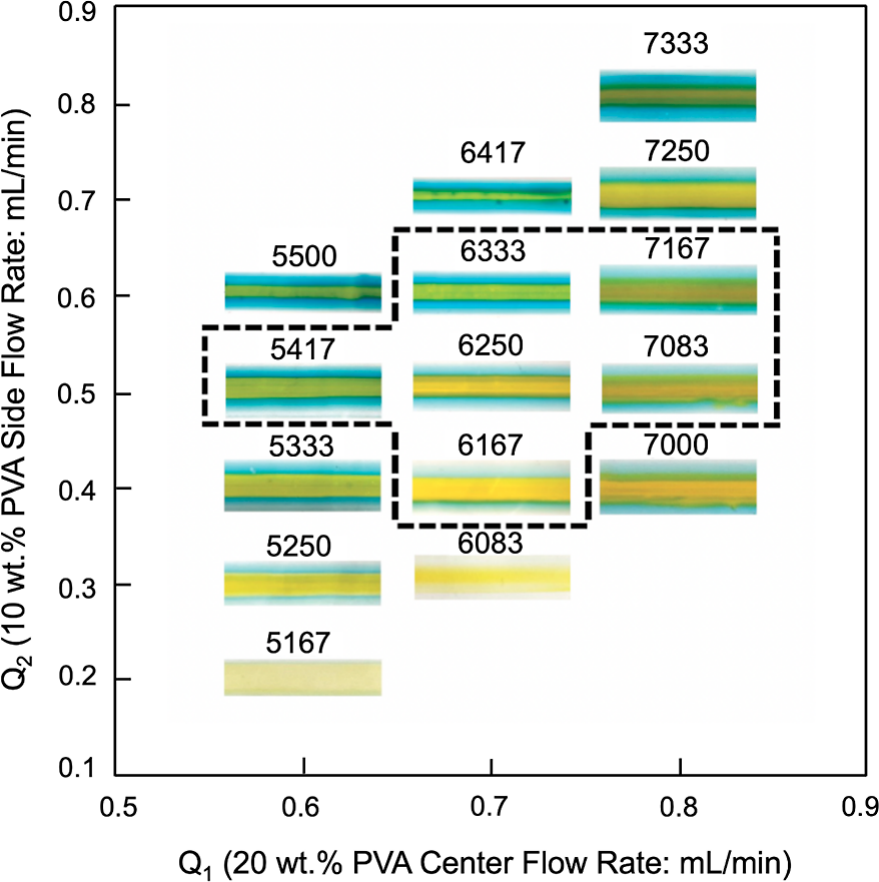
Quality chart showing coating at different ranges of flow rates both at the lower and upper Pe limits.

The effect of the flow rate on the quality of the gradient thin film is illustrated in Figure 1. Here, 20 wt % yellow PVA (center flow stream) and 10 wt % blue PVA (side flow streams) were used. To understand the effect of changing the flow rate, either the flow rate of the center flow stream or the side flow streams were held constant, while the other was changed in increments of 0.1 mL/min. As shown in Figure 1, gradient structures were formed; however, the flow rate impacts the width and the thickness of the coated film. For Pe values between 5000 and 15,000, the center flow rate ranges from 0.6 to 0.8 mL/min, while the side flow rate ranges from 0.3 to 0.5 mL/min, as shown by the solid lined box in Figure 1. When the Pe value is lower than 5000 (e.g., *Q*_1_ = 0.6 mL/min and *Q*_2_ = 0.6 mL/min), irregular coating occurs, whereas when Pe is greater than 15,000 (e.g., *Q*_1_ = 0.8 mL/min and *Q*_2_ = 0.4 mL/min) the center flow dominates the overall film. These results align with what has been shown previously^44^ in which the gradient formation of two fluids is formed at Pe values between 5000 and 15,000. Following this, the gradient formulation at the deposition phase is explored in Figure 1, which shows the effect of the Pe value on the width of the overall gradient structure.

**Figure 2:**
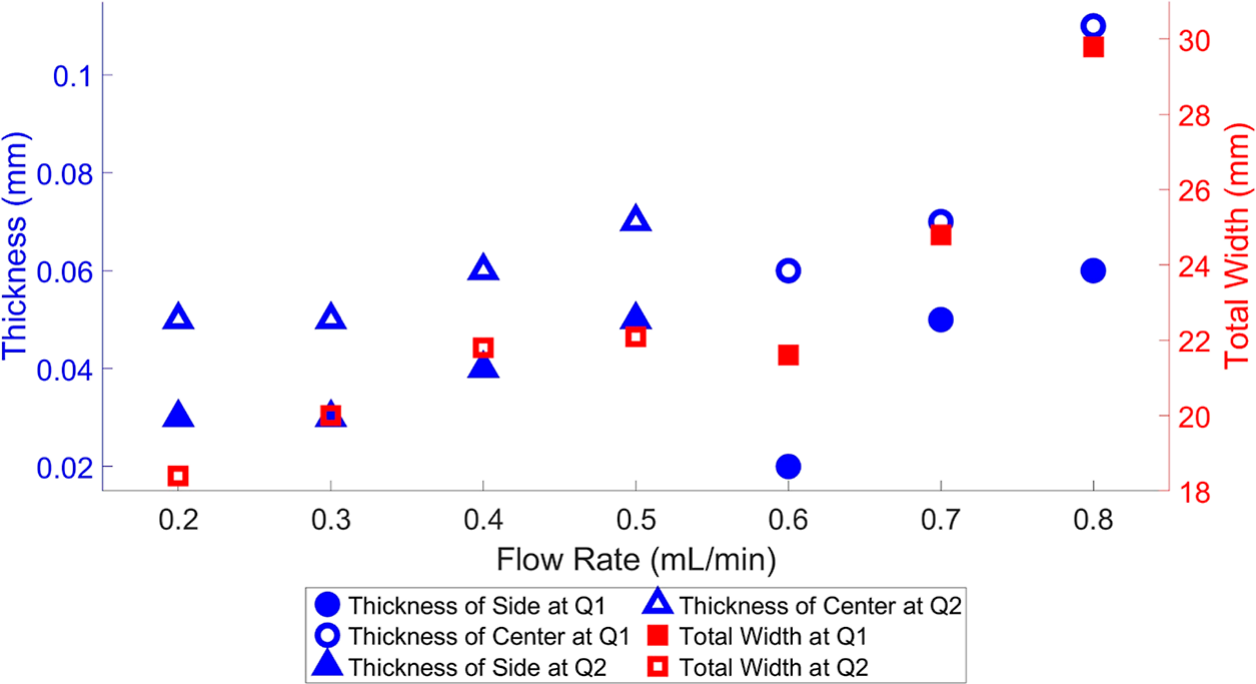
Effect of change in the flow rate to the dimension of the gradient film, where in blue the thickness of both the center and sides when the center flow rate is altered while the side flow rate remains constant and vice versa is illustrated, and in red the total width when the center flow rate is altered but the side flow is constant and vice versa.

To examine the impact of the center flow rate on the dimensions of the gradient film, the side flow rates were kept constant during the fabrication of the gradient film. Similarly, to explore the influence of side flow rates, the center flow rate was held constant during fabrication of the gradient film. The thickness and width of each coated region are compared in Figure 2. For both thickness and width, they have a positive correlation with the flow rate as more flow will result in more spreading as the coating gap is constant. From the slope differences of different *Q*_1_ and *Q*_2_, it is noticed that alteration in the center flow rate had more impact on the mixing mechanism with a wider region of gradient regions and a center region, as shown in Figure 2. The measured width and thickness values all had errors of less than 3% across multiple experiments; each experiment was conducted at least four times.

The change in the side flow rate had an impact on mixing and the overall geometry of the gradient structure, but compared to the change in the center flow rate the impact is marginal, as shown in Figure 2. This phenomenon happens because viscosity only affects the center flow rate, based on eq 1. Hence, as the viscosity ratio between the two fluids increases, it is likely that there will be more mixing as the particles tend to move from a high concentration (more viscous) to a low concentration (less viscous). Since there is a relatively large volume of fluid being coated into a constrained free boundary, spreading was inevitable due to mass conservation. Parsekian et al.^59^ formulated some empirical formulas that can predict the overall width of the coated film using the dimensions of the slot die and the coating parameters. The dimensional analysis starts with eq 2 shown below to calculate the line-of-fit represented by Π_a_
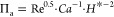
2where Re is the Reynolds number and H* represents the dimensionless length ratio at the die lip. Re has been modified by Parsekian^59^ et al.,^54^ as shown in 3 and H* is also shown in eq 4.
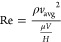
3
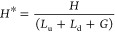
4where *v*_avg_ is the average flow velocity followed through to the die lip represented by eq 5. In eq 4, *L*_u_ is the upstream die lip length, *L*_d_ is the downstream die lip length, and *G* is the thickness of the shim, which are shown in Figure S2.
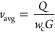
5where *Q* is the flow rate and *w*_c_ is the outlet width. To implement the experimental data into a linear regression, a piecewise empirical model is formed for the advancing fluids as shown below
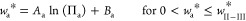
6a
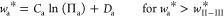
6bwhere *w*_a_^*^ is the dimensionless width shown as *w*_a_^*^w_a_/*w*, and *w*_II–III_^*^ is the range dimensionless width when the pinning starts to occur. Initially, *w*_II–III_^*^ is set to (*L*_u_ + *L*_d_ + *G*)/*w*. Note that only advancing calculations are investigated for this paper as it is assumed that the film widths created are based on a steady-state nonstopping system.

By implementing the given variables from eqs 2–5 for changing the *Q* value, two piecewise equations for each case can be solved using the measured *w* values stated above. As a result, eqs 6a and 6b are derived following their given conditions. For the case when *Q*_1_ is 0.8 and *Q*_2_ is 0.5 mL/min, the regression equation is shown in eq 7.
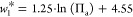
7

Using the derived equation, the theoretical total width is calculated to be 29.62 cm with an error between the experimental value of 1.28%. This implies that the spreading phenomenon at the deposition is expected to occur due to the high flow rate following mass conservation.

#### 3.2.2 Functionally Gradient Thin Films

Functionally gradient thin films were formed using R2R to illustrate that the gradients formed on the film can exhibit multifunctionality (e.g., active or inactive regions). In a recent paper published by Parsekian and Harris,^39^ it was shown that two miscible materials can be codeposited simultaneously using slot die coating, even if one fluid is wetting and the other is nonwetting. It should be noted that a nonwetting fluid generally poses significant challenges when coating, requiring changes in fluid chemistry or surface modifications to the substrate. However, their work shows that the wetting fluid can stabilize or scaffold the nonwetting fluid, allowing both materials to be codeposited on an R2R system without the need for these modifications to create distinct coating lines with very little mixing.^39^

Following this discovery, gradients of a functional material, e.g., nonwetting conductive polymers stabilized with PVA were fabricated and analyzed. It should be noted that typically PEDOT:PSS is altered with surfactants or other additives prior to coating to enhance its wettability.^60−62^ In this work, the functional materials serve as the center fluids for the samples. However, in agreement with what was shown by Parsekian and Harris,^30^ gradient thin films were achievable without the need for such alterations. It is believed that the wetting fluid, i.e., PVA, acts as a wetting enhancer or scaffold, which aids the film-coating stability of the nonwetting fluid. As the nonwetting fluid (PEDOT:PSS or PEI) is coated in-between two side wetting fluids (PVA solution), the side fluids tend to create a wall-like barrier which prevents the further horizontal spreading of the center fluid. The equilibrium formed at the interfacial tension between the non-wetting fluid and the wetting fluid allows for such phenomena to occur. Young’s equation^63^ is shown in eq 8, where *γ*_*SG*_ is the surface tension at the solid–gas phase, *γ*_*SL*_ represents the surface tension at the solid–liquid phase, *γ*_*LG*_ represents the surface tension at the liquid–gas phase, and θ represents the contact angle. This equation also stands for between the immiscible liquid–liquid interface instead of gas as shown in 9 where γ_L_1_L_2__ represents the interfacial tension between two liquids, γ_SL_1__ represents the surface tension of solid–liquid 1, and γ_SL_2__ represents the surface tension of solid–liquid 2. As two immiscible liquids are in contact with each other, it can be assumed that the interfacial surface tension is very small.^64^ Therefore, the interface between nonwetting and wetting liquids are at equilibrium and stable, which allows for the wetting of nonwetting liquids when they are codeposited with the wetting fluids.^39^
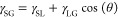
8
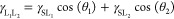
9

**Figure 3:**
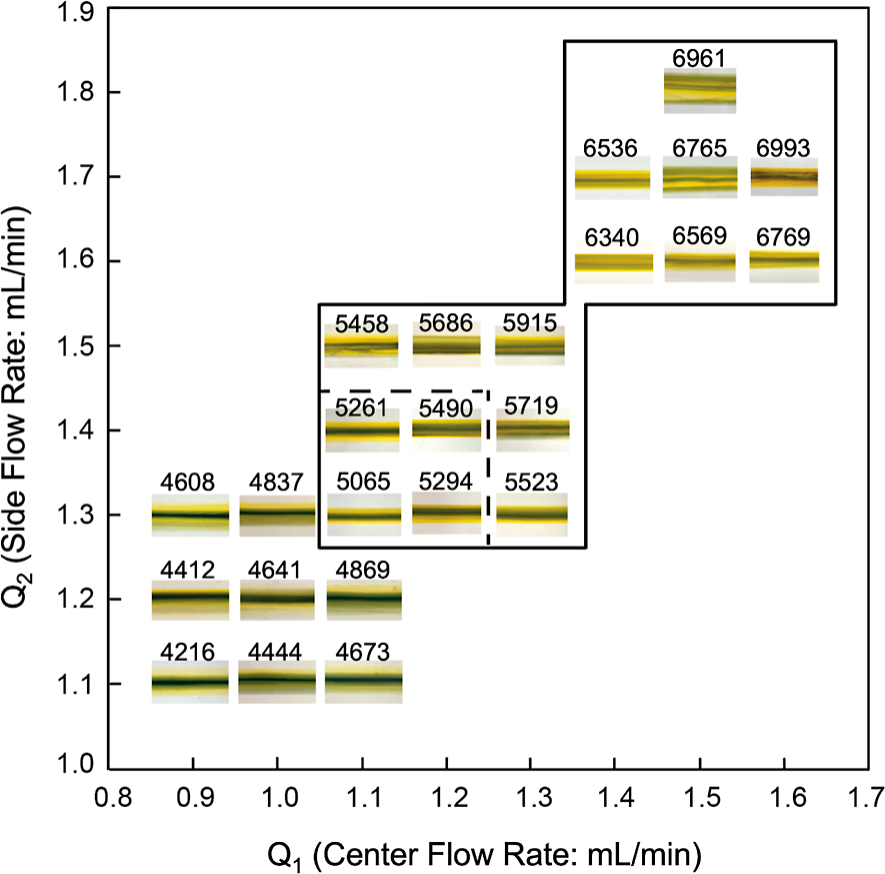
Quality chart showing coating at different ranges of flow rates at both the lower and upper Pe limits.

A parametric study was performed such that the flow rates exceed both the lower and upper limits of the Pe values where mixing is induced. This study allows for a comparison of the theoretical and experimental values. Although the flow rate ranges are deduced to have different possible working conditions theoretically (at which Pe is over 5000), as shown in the solid line box region of Figure 3, only four combinations, with *Q*_1_ ranging from 1.1 to 1.2 [mL/min] and *Q*_2_ ranging from 1.3 to 1.4 [mL/min], had quality gradient formation where a clear color scheme is noticeable, as shown in the dashed box of Figure 3. Conversely, at flow rate combinations below the lower Pe limit, the center flow shows eroded wavy patterns, and beyond the upper Pe limit, the center flow dominates the film structure.

For the mixing to occur, the center flow needs to have a higher viscosity compared with the side viscosity. Unfortunately, PVA solutions with higher than 7.5 wt % had a higher viscosity than PEDOT:PSS, so mixing would not occur. Less viscous PVA resulted in a coating defect known as dripping, which is an overflow of the liquid during coating. Changing the viscosity ratio was not feasible, since the viscosity of the PEDOT:PSS could not be changed. Although some combinations of Pe are within the calculated range, gradients did not form when the center flow fluid dominates. Moreover, the region composed of PEDOT:PSS is very narrow (1–3 mm of the PEDOT:PSS, 5–9 mm of the center width containing the mixed region, and 12–24 mm of the total width) compared to that of 20 wt % PVA/10 wt % PVA gradient films (4–12 mm of the center width containing the mixed region and 11–28 mm of the total width), regardless of the concentration. Such phenomenon occurs due to the significantly low *D* of PEDOT:PSS. Therefore, it is likely that the 20 wt % PVA/10 wt % PVA concentrations are interdiffusing as the *D* of PVA is high compared to that of PEDOT:PSS. Whereas, for the PEDOT:PSS/7.5 wt % PVA gradient thin film, 7.5 wt % PVA diffuses into PEDOT:PSS. As previously mentioned, the widths of each region exceed the outlet width due to the high inlet flow rates following the mass conservation, as described in Section 3.2.1.

**Figure 4:**
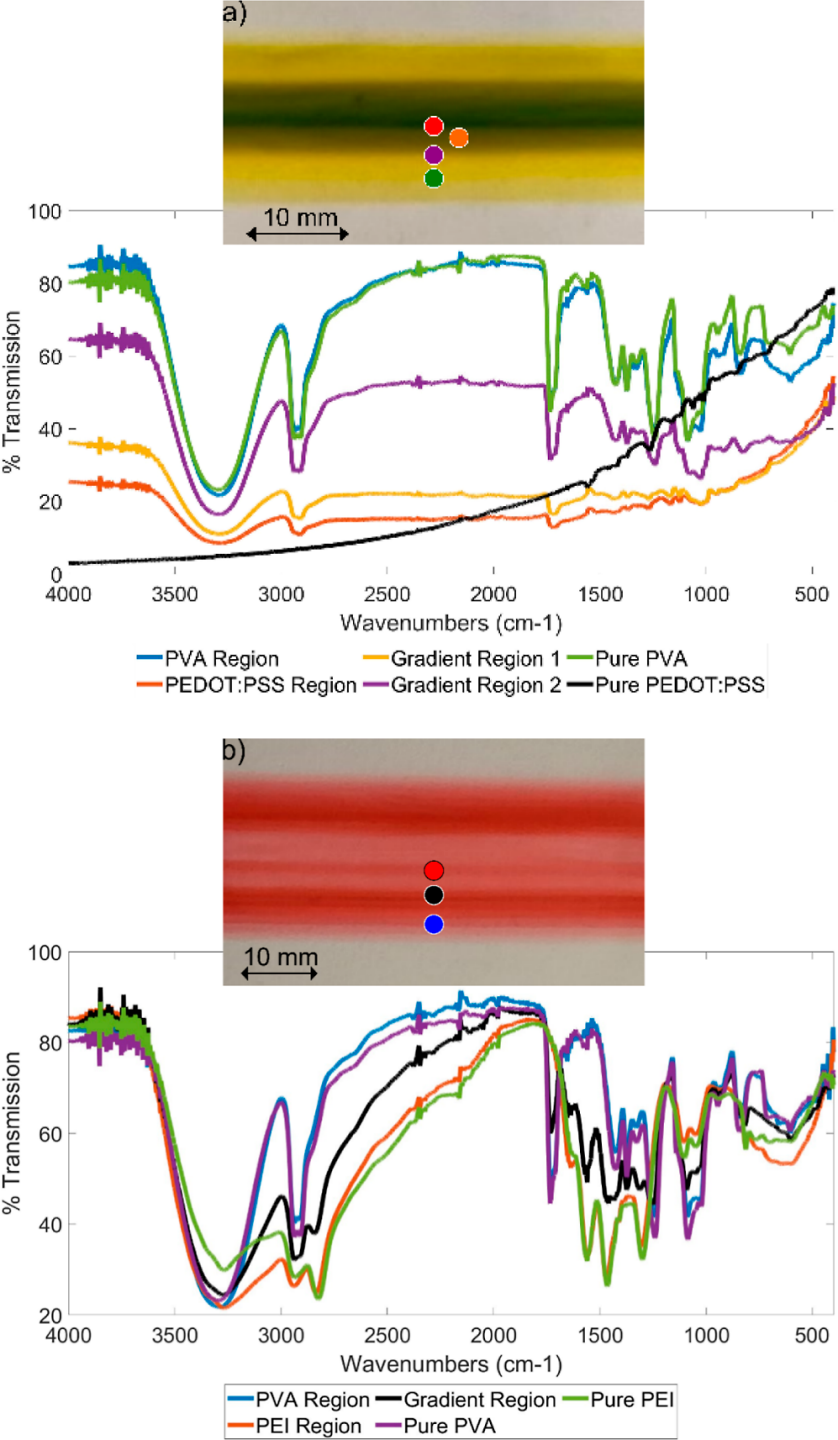
Functionally gradient thin films and the FTIR results of (a) PEDOT:PSS in the center and 7.5 wt % PVA as the side fluids and (b) 25 wt % PEI in the center and 10 wt % PVA along the side.

Comparing PEDOT:PSS/7.5 wt % PVA (yellow), coated at flow rates of 1.1 and 1.4 mL/min, respectively, to 25 wt % PEI/10 wt % PVA (red), coated at flow rates of 1.0 and 0.8 mL/min, respectively, it is observed that the mixing is more prominent for the 25 wt % PEI/10 wt % PVA (red), as shown in Figure 4a,b. It is evident by the pink hue that the red food dye gets significantly mixed into the clear PEI, resulting in a much higher gradient region. While the same may be true for the PEDOT:PSS, as mentioned previously, due to the difference in the *D*, the region of gradient formation is comparatively narrow. The percent transmission measured using FTIR at different regions of the formed gradient film is compared with that of pure PVA and PEDOT:PSS. As shown in the resultant graph in Figure 4a, the transmission peaks in the PVA region of the film are close to that of pure PVA, and the peaks in the PEDOT:PSS region from the film is not exact but has similar peaks to pure PEDOT:PSS. This is mainly because the PEDOT:PSS region in the gradient film is very narrow, so that the measured region was smaller than the FTIR measuring pointer. However, the results still show that the peaks from the FTIR look more like that of PEDOT:PSS as the measuring region moves from PVA to PEDOT:PSS on the gradient film. For Figure 4b, the FTIR measurement clearly shows that PEI and PVA regions have similar peaks to their pure materials’ peak, and the gradient region has peaks in-between the peaks of the two regions. This is a good indication of the formation of gradient structure formation.

#### 3.2.3 Effect of Mixing

**Figure 5:**
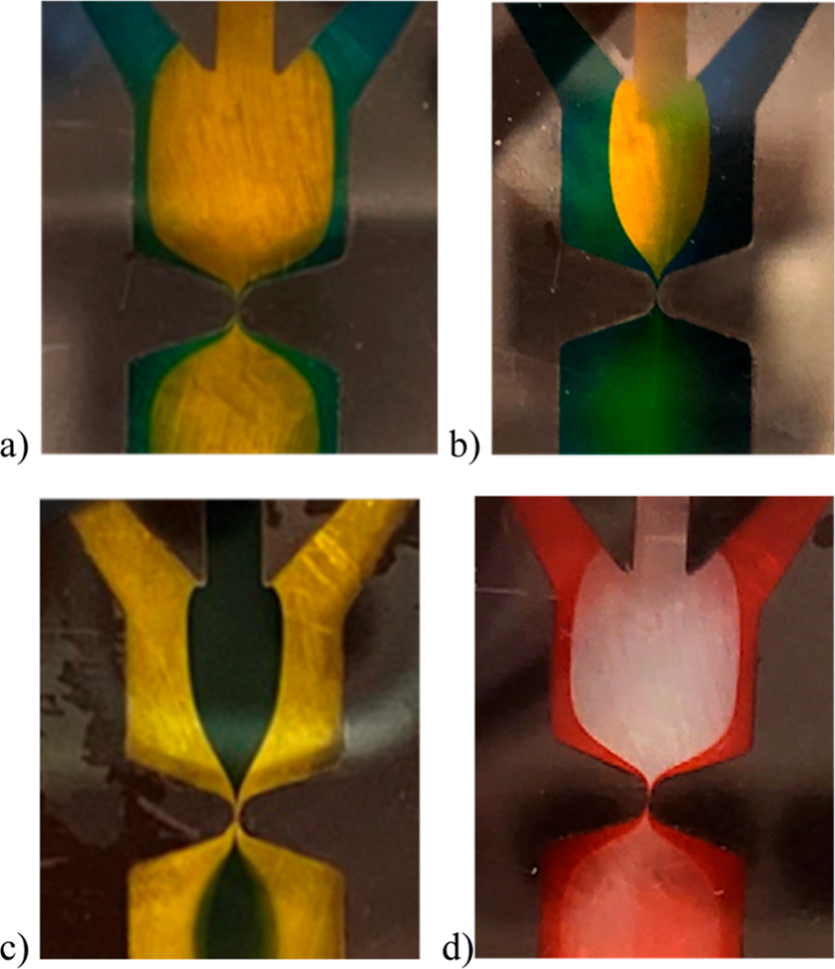
Internal geometry of the microfluidic channel for the four samples discussed: (a) 20 wt % PVA and 10 wt % PVA, (b) 15 wt % PVA and 10 wt % PVA, (c) PEDOT:PSS and 7.5 wt % PVA, and (d) 25 wt % PEI and 10 wt % PVA.

It is observed that while the functional materials can be fabricated, the level of mixing between the fluids changes significantly as a function of χ, the viscosity ratio between the center fluid and the side fluid where the center fluid has a higher viscosity. Based on the pictures shown in Figure 5, qualitatively, as χ increases, the mixing of the fluids after passing through the first microchannel increases because a higher χ means more advection or transport of fluids. The mixing of the two fluids is greater as seen in Figure 5b,d which have χ values of 20 and 10, respectively. The mixing is less for a smaller χ value as shown in Figure 5a,c which are 5 and 2.667, respectively. The differences in the colors after the fluids pass through the microchannel at large and small χ values are clear.

### 3.3 Characterization

#### 3.3.1 SEM Imaging

**Figure 6:**
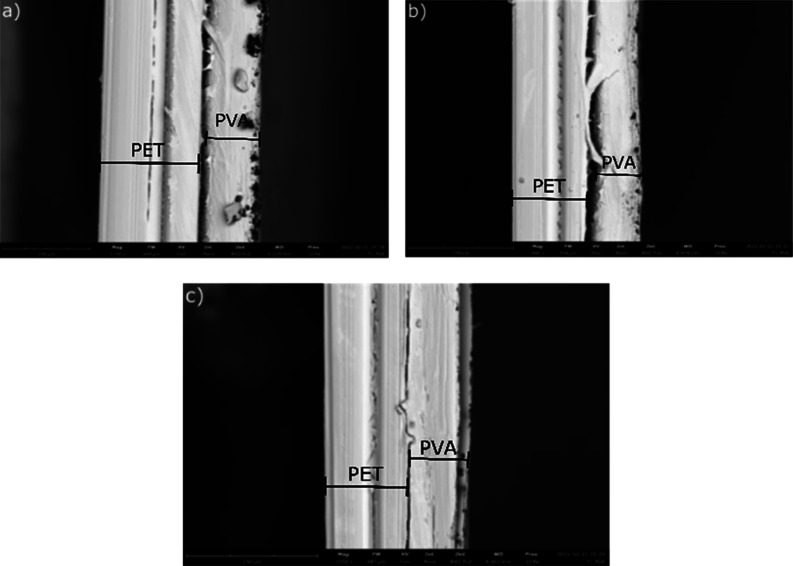
Cross-sectional image of the PVA/PVA gradient film with the (a) center region, (b) side region, and (c) gradient region.

The cross-sectional images shown in Figure 6 depict the thickness difference along the gradient structure. For the 20 wt %/10 wt % PVA gradient film, the thickness of center, side, and gradient regions is 68.70, 51.32, and 55.72 μm, respectively, as shown in Figure 6. Using SEM, not only the thickness is determined but also the physical evidence of the gradient formation. While it lacks color determination, through the gradual increase in thickness from the side region of the film to the center region, gradient formation can be inferred.

#### 3.3.2 UV–Vis Spectroscopy

**Figure 7:**
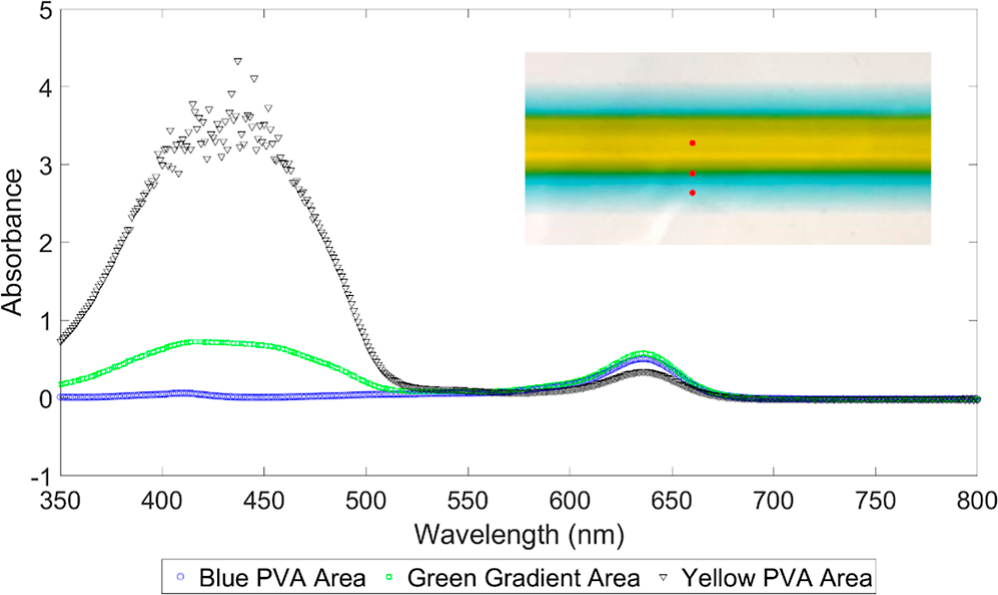
UV–vis spectra of 20 wt % PVA at the center (yellow), 10 wt % PVA at the sides (blue), and the gradient of 20 wt %/10 wt % PVA (green). Approximate measured regions are marked with red dots.

A UV–vis spectrometer was used to assess the gradient structure based on the absorbance peaks of each pigment, as shown in Figure 7. As an example, a 20 wt %/PVA 10 wt % PVA gradient thin film, where *Q*_1_ = 0.7 mL/min and *Q*_2_ = 0.5 mL/min, is considered. The characterized sample is shown as an inset in Figure 7, where the 20 wt % PVA (yellow) is shown along the center, 10 wt % PVA (blue) is shown along the sides, and green shows the gradient region, i.e., concentrations of 20 wt %/10 wt % PVA. The peak absorbance for the blue pigment is at a wavelength of approximately 630 nm^65^ and approximately 405 nm^66^ for the yellow pigment. As shown in Figure 7, the absorbance peaks for the blue and yellow regions follow the known wavelength values, and in the gradient region both peaks are apparent; thus, both pigments coexist in this region. It can be observed that a small amount of blue pigment diffuses into the center fluid because at higher Pe values the side fluid begins to mix with the center fluid. It should be noted that the scattering illustrated in the yellow absorbance peak is due to experimental error.

#### 3.3.3 Adhesion Test

**Figure 8:**
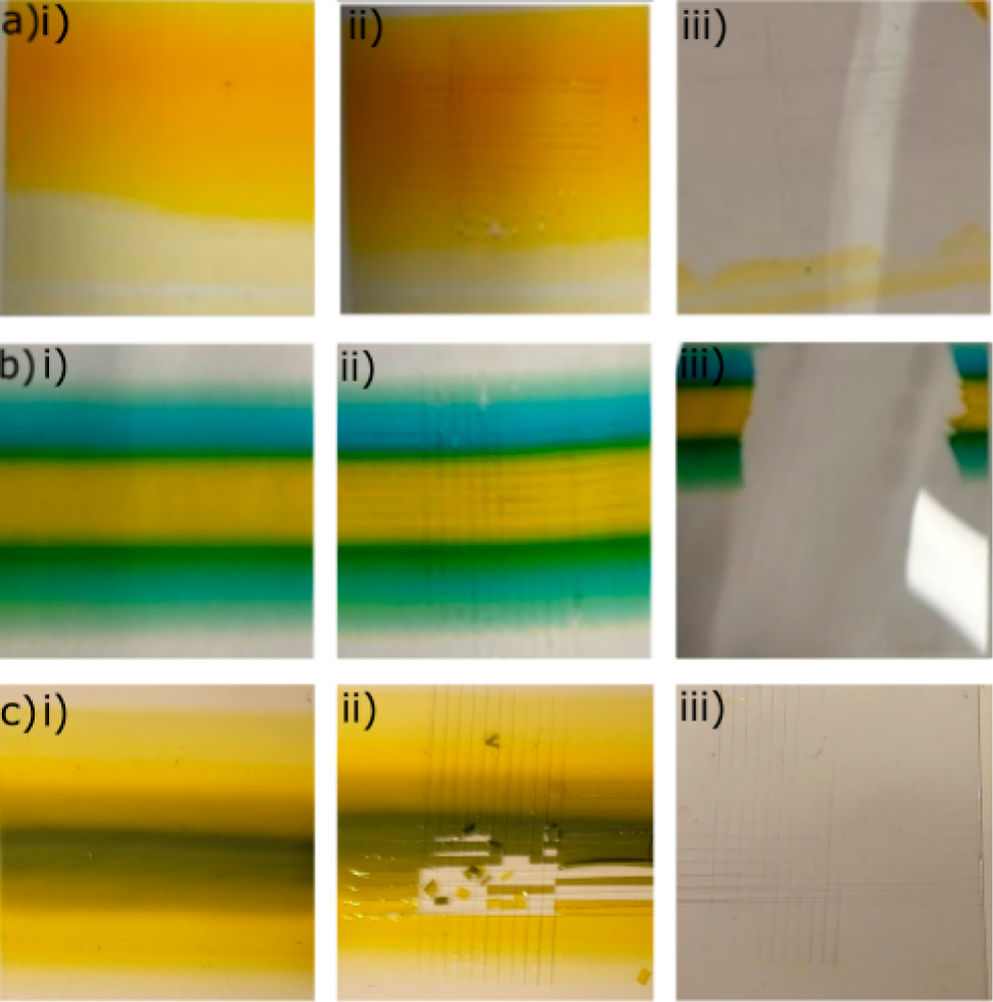
Adhesion test results of (a) 10 wt % PVA thin film, (b) 20 wt %/10 wt % PVA gradient thin film, and (c) PEDOT:PSS/7.5 wt % PVA gradient thin film. (i–iii) Original, cross-cut, and tested samples, respectively.

To understand the adhesion of various samples, five samples were tested, including 10 wt % PVA, 20 wt % PVA/10 wt % PVA gradient thin film, PEDOT:PSS/7.5 wt % PVA gradient film, 25 wt % PEI/10 wt % PVA gradient film, and fully blended 25 wt % PEI/10 wt % PVA, as illustrated in Figure 8a(i)–e(iii). For each set of data, the unscratched (as-fabricated) samples are shown in the first column, the scratched samples are shown in the second column, and the tested samples (scotch tape pull test) are shown in the third column.

10 wt % PVA serves as a control, although other concentrations of PVA were used in the study. It is believed that 10 wt % PVA is representative of the behavior that would be exhibited by other PVA concentrations. It was observed that 10 wt % PVA has very poor adhesion on PET, as illustrated in Figure 8a(i)–(iii). 10 wt % PVA on PET exhibits category 0B delamination, meaning complete (100%) or near complete removal of the PVA from the PET surface, as exhibited in Figure 8a(iii). Although it is not present, the experiments have been performed with pure, noncolored PVA to investigate the effect of color dyes, and it has been shown that addition of colors did not have any effect on the adhesive property.

A 10 wt % PVA/20 wt % PVA gradient thin film was made and assessed, as shown in Figure 8b(i)–(iii). It has been further observed that regardless of PVA concentration, e.g., 10–20 wt %, since the gradient region would be some concentration between 10 and 20 wt % PVA, the adhesive forces of the PVA on PET are not improved, as demonstrated in Figure 8b(iii). Hence, in the case of PVA/PVA gradient thin films, the structure had no improvement or added advantage when fabricated as a gradient structure.

The tested PEDOT:PSS/7.5 wt % PVA gradient film is shown in Figure 8c(i)–(iii). It is observed that the PEDOT:PSS delaminated during the scratching stage, as shown in Figure 8c(ii). The overall assessment is an evaluation of 0B, complete delamination of the PEDOT:PSS/7.5 wt % PVA gradient structure, as shown in Figure 8c(iii). One of the main reasons PEDOT:PSS/7.5 wt % PVA delaminates from the negatively charged PET surface is because PVA is nonionic and PEDOT:PSS is negatively charged, thus there is no substantial force to keep the materials adhered to one another.

**Figure 9:**
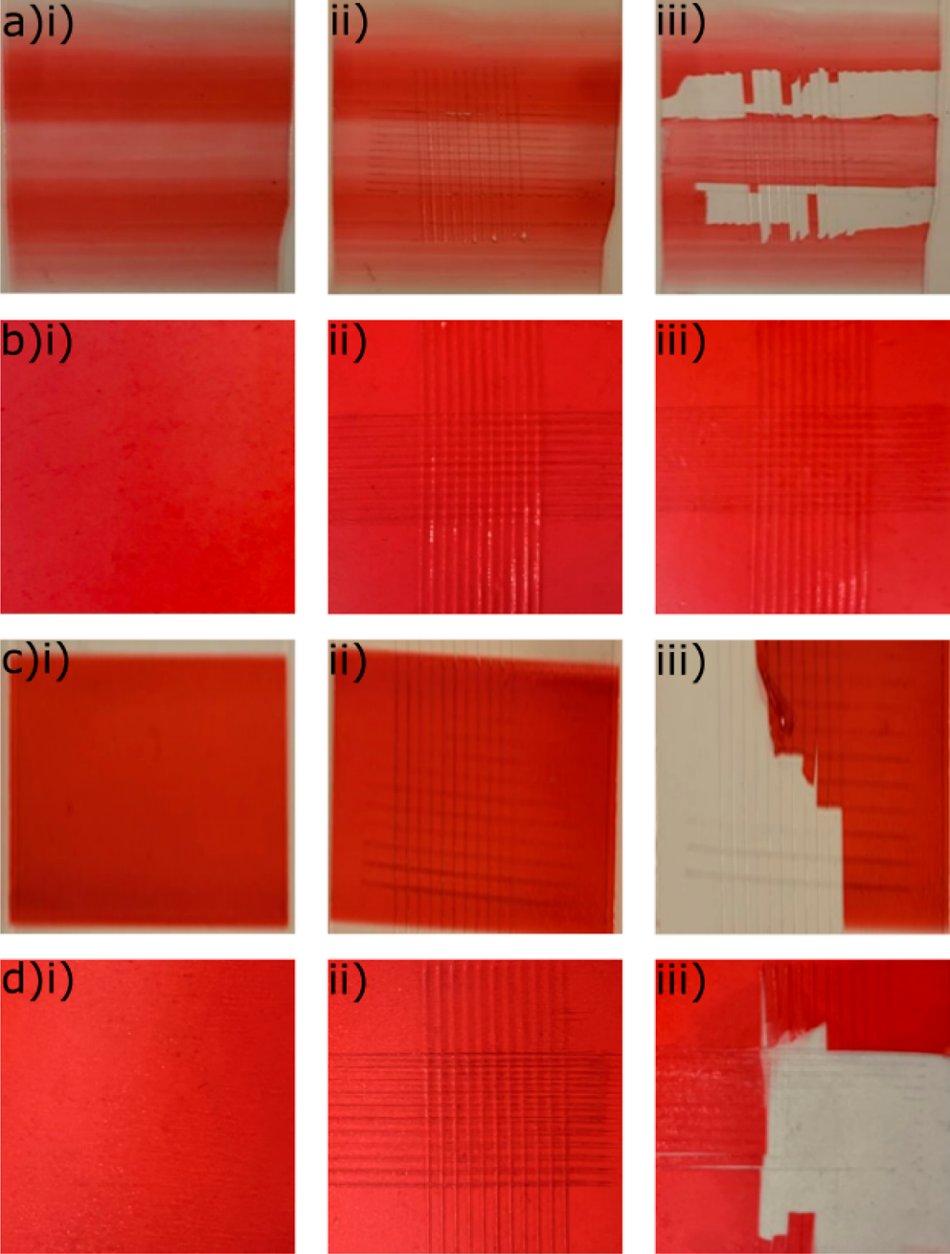
Adhesion test results of (a) 25 wt % PEI/10 wt % PVA gradient thin film, (b) fully blended 2:1 ratio of 25 wt % PEI/10 wt % PVA thin film, (c) 1:1 ratio of 25 wt % PEI/10 wt % PVA thin film, and (d) 1:2 ratio of 25 wt % PEI/10 wt % PVA thin film. (i–iii) Original, cross-cut, and tested samples, respectively.

As both PVA and PEDOT:PSS have very poor adhesion to PET, the improvement of adhesion due to the gradient structure could not be proven. Therefore, a well-known adhesive polymer, PEI, was used. A gradient thin film of 25 wt % PEI and 10 wt % PVA was tested, as shown in Figure 9a(i)–(iii). Interestingly, this gradient structure had mixed delamination results. As illustrated in Figure 9a(iii), the 25 wt % PEI is category 5B (0% delamination) and only the PVA delaminated is category 0B (100% delamination). Furthermore, the gradient region exhibited very high adhesion to the PET surface also obtaining a category 5B assessment even with the presence of PVA. It is well known that positively charged beads have higher adhesive forces on negatively charged surfaces.^67^ Since PEI is positively charged and PET film is negatively charged, the high adhesion force of 25 wt % PEI on PET is expected.

25 wt % PEI and 10 wt % PVA solutions were fully blended in 2:1, 1:1, and 1:2 weight ratios to also prove the efficiency of the gradient structure, as shown in Figure 9b–d. From Figure 9b(i)–(iii), it can be seen that the fully blended 2:1 ratio of 25 wt % PEI and 10 wt % PVA solution film showed no sign of delamination or 5B classification. It is observed that in 1:1 and 1:2 weight ratio 25 wt % PEI and 10 wt % PVA solutions, approximately 100% of the blend film is delaminated, thus belonging to category 0B. Compared to the PEI/PVA gradient structure, which had less delamination at the gradient region, the blended PEI/PVA solution delaminated completely. This may have occurred because of different concentrations of PEI in the gradient structure, and it can be inferred that there are more than 50% PEI in the gradient regions. For the future work, different concentrations of PEI/PVA blends could be observed to verify the minimum PEI percentage that improves the adhesive properties.

#### 3.3.4 Conductivity Test

**Figure 10:**
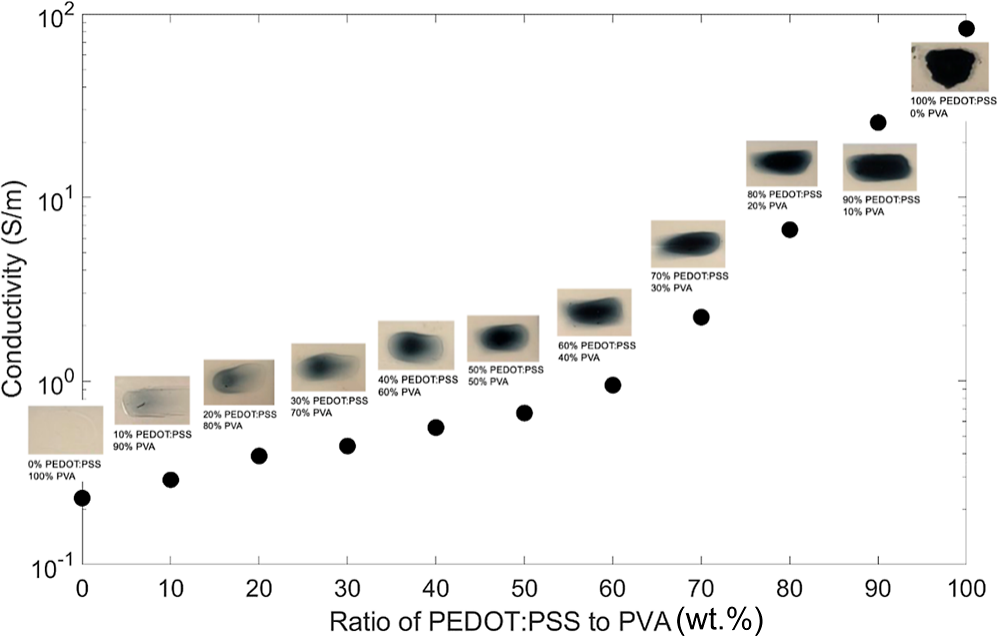
Electrical conductivity at different PEDOT:PSS/PVA ratios.

To determine the electrical conductivity for gradient blends of PEDOT:PSS/7.5 wt % PVA, droplets of varying ratios of blade-coated PEDOT:PSS/7.5 wt % PVA mixtures were tested to measure the electrical conductivity of the range of materials from neat to ratios of 10–90 wt %, as shown in Figure 10. This data can be used to verify the existence of a gradient film as well as aid with a quantitative assessment of the overall material concentrations within the measured regions. The PEDOT:PSS/PVA blends have been drop-cast on the PET for this exploration. The thickness of each drop, which was spread to about 12.5 mm, was measured using a micrometer. The coatings had a uniform thickness of roughly 0.03 ± 0.001 mm, regardless of the weight ratio; however, the widths were not uniform. Based on literature, the conductivity of PEDOT:PSS ranges from 20 to 100 S/m,^68^ and the PVA film has a conductivity of 0.248 S/m.^69^ As shown in Figure 10, the conductivity of droplets of pure 7.5 wt % PVA, PEDOT:PSS/7.5 wt % PVA blends, and PEDOT:PSS as purchased, ranged from 0.22 to 83.3 S/m, which align with the expected conductivity values. The electrical conductivity of PVA/PEDOT:PSS blends has been found to increase in previous work,^70^ as well. In this case, the conductivity within the gradient structure from the previous film is close to that of 80% PEDOT:PSS/7.5 wt % PVA solution with a value of 6.67 S/m. These results of conductivity are a significant improvement on those reported by Liu et al.,^71^ for the electrospun fully blended mixture of PEDOT:PSS and PVA solutions. Their samples exhibited significantly lower conductivity, ranging from 0.00048 to 0.0017 S/m for thin films that were about 250 nm thick. Here, the gradient structure produces a higher electrical conductivity compared to that of the fully blended materials. This is because the gradient structures have the functional molecules dispersed in order, whereas fully blended materials have the functional molecules dispersed randomly. This allows for the functional molecules to react more readily to each other.^72−75^ This can insinuate that gradient structures, which expect 50% mixing at the center, are more efficient as it produces the same result as that of 80% PEDOT:PSS. Such efficiency reflects that by continuously fabricating conductive thin films, less functional materials are needed.

#### 3.3.5 FTIR Spectroscopy

**Figure 11:**
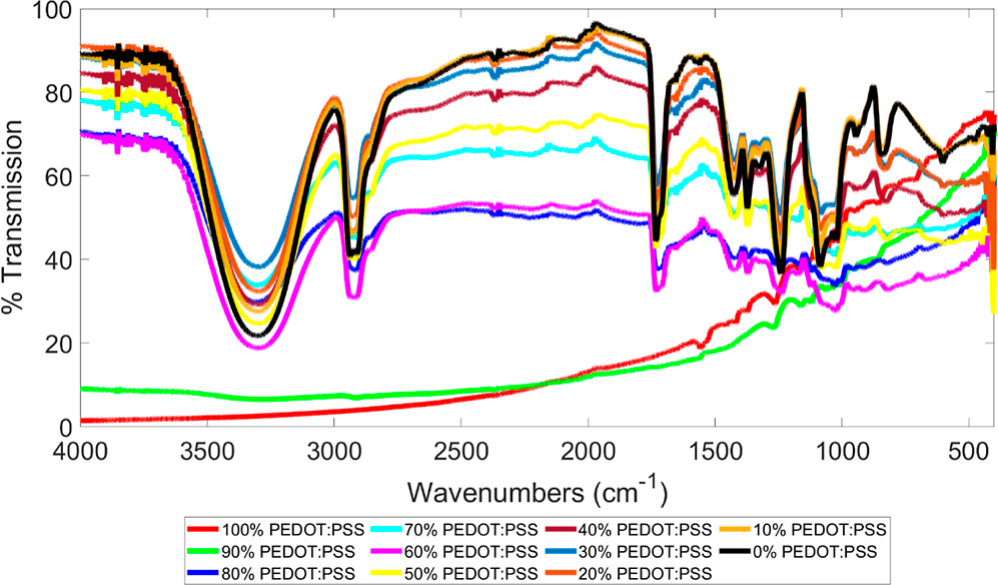
FTIR results of different ratios of PEDOT:PSS/PVA.

Earlier, FTIR was utilized to verify the presence of gradient structure in Figure 4. An in-depth investigation was performed with FTIR to compare the PEDOT:PSS/PVA gradient structure with fully blended PEDOT:PSS/PVA solutions. As shown in Figure 11, the PEDOT:PSS/10 wt % PVA blends follow the FTIR curves of PVA, when the ratio of PEDOT:PSS to PVA is at or below an 80% ratio. As the ratio increases above 80%, the FTIR peak patterns start to follow those of PEDOT:PSS. This trend is also present for FTIR results of the gradient structure formed between the PEDOT:PSS and 10 wt % PVA. In the PEDOT:PSS region, peaks for PVA are present, which shows that at below 80 wt % PEDOT:PSS to PVA ratio, the PVA is notably present, meaning that the maximum mixed region would be considered as 80 wt %.

**Figure 12:**
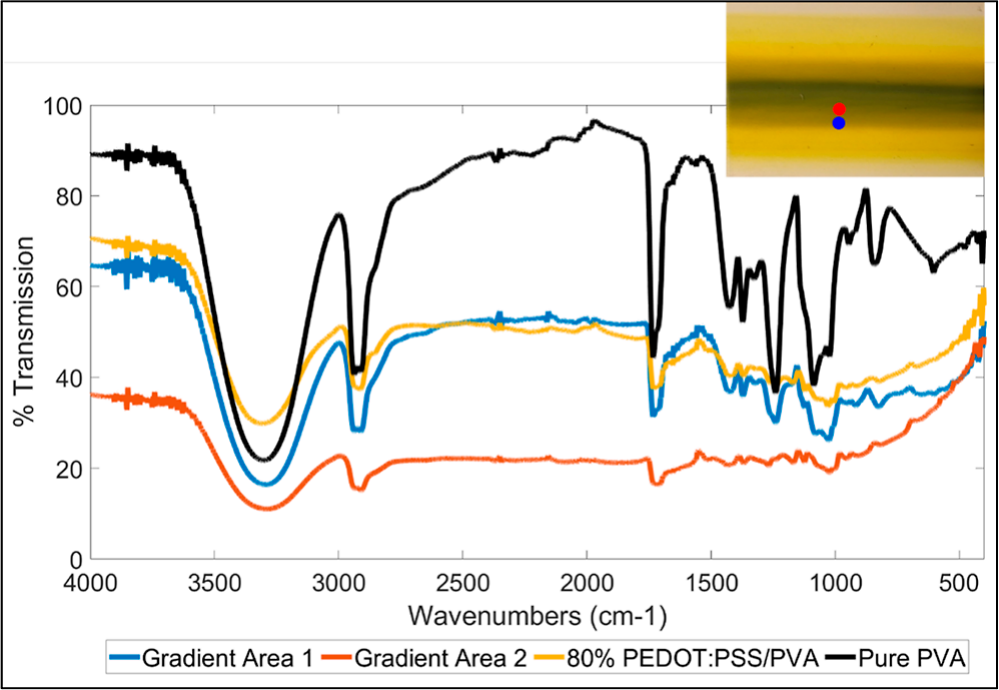
FTIR results to compare the 80% PEDOT:PSS/PVA mixture, pure PVA, and the two gradient regions (closer to PEDOT:PSS and closer to PVA).

In Figure 12, Gradient Area 1 represents the gradient region closer to 10 wt % PVA and Gradient Area 2 represents the gradient region closer to PEDOT:PSS. The FTIR results show that the transmission decreases as the PVA contents decrease, which is expected as the content of PVA also decreases. It is shown in the FTIR graph that the 80% PEDOT:PSS/PVA mixture has similar trends as materials with higher ratios of PEDOT:PSS. Given the trends of the FTIR data, the similarities of the electrical conductivity for the gradient structure and 80% PEDOT:PSS/PVA are reasonable.

## 4 Conclusions

In this work, it has been shown that the gradient can be formed instantly and be coated using slot die coating on an R2R system. By controlling the coating parameters will allow the two materials to mix to form a gradient, which was validated with SEM and FTIR analyses. Functional gradient structures were investigated and were found to have enhanced properties within the gradient, although there are limits depending upon the ratios or concentrations of the fluids. It was found that material structures composed of conductive (PEDOT:PSS) and nonconductive (PVA) fluids are semiconductive within the gradient region. The electrical conductivity was found to be the same as the 80% ratio PEDOT:PSS/7.5 wt %. PVA structure. While beyond the scope of this work, grazing-incidence wide-angle X-ray scattering^76^ may be used to further characterize conductive materials, such as PEDOT:PSS. Using PEI, it was found that the adhesive property of a nonwetting fluid (e.g., PVA) can be enhanced along the gradient structure when a wetting fluid (e.g., PEI) was used. Fully blended PEI/PVA was not found to have improved adhesive properties. This work shows that slot die coating on a R2R basis can be used to fabricate gradient thin films that exhibit multiple properties across the film.

## Supporting Information Available

Chemical structure of used fluid materials, schematic of the overall experimental setup, and the shim geometry design (PDF)

## References

[ref1] Naserifar N.; LeDuc P.; Fedder G. Material Gradients in Stretchable Substrates Toward Integrated Electronic Functionality. Adv. Mater. 2016, 28 (18), 3584–3591. 10.1002/adma.201505818. 26989814

[ref2] Xu Y.; Yang Y.; Yan D.-X.; Duan H.; Zhao G.; Liu Y. Gradient Structure Design of Flexible Waterborne Polyurethane Conductive Films for Ultraefficient Electromagnetic Shielding with Low Reflection Characteristic. ACS Appl. Mater. Interfaces 2018, 10 (22), 19143–19152. 10.1021/acsami.8b05129. 29766720

[ref3] Estelle K.; Blair D.; Evans K.; Gozen B. Manufacturing of Smart Composites with Hyperelastic Property Gradients and Shape Memory Using Fused Deposition. J. Manuf. Process. 2017, 28 (3), 500–507. 10.1016/j.jmapro.2017.04.018.

[ref4] Baker B.; Trappmann B.; Stapleton S.; Toro E.; Chen C. S. Microfluidics Embedded Within Extracellular Matrix to Define Vascular Architectures and Pattern Diffusive Gradients. Lab Chip 2013, 13, 3246 10.1039/c3lc50493j. 23787488 PMC4082768

[ref5] Kim J. H.; Kim H. J.; Jeon J. G.; Shin G.; Lee J.; Yun S.; Kang T. J. Temperature Gradient-Driven Multilevel and Grayscale Patterning of Tosylate-Doped Poly(3,4-Ethylenedioxythiophene) Films for Flexible and Functional Electronics. Adv. Mater. Technol. 2021, 6 (10), 2100613 10.1002/admt.202100613.

[ref6] De France K.; Hoare T.; Cranston E. Review of Hydrogels and Aerogels Containing Nanocellulose. Chem. Mater. 2017, 29, 4609–4631. 10.1021/acs.chemmater.7b00531.

[ref7] Moonprasith N.; Tatsumichi M.; Nakamura K.; Kida T.; Tsubouchi K.; Hiraoka T.; Yamaguchi M. Preparation of Graded Materials for Miscible Polycarbonate/Poly(Methyl Methacrylate) Blends by Segregation Under Shear Flow. J. Appl. Polym. Sci. 2022, 140, 53258 10.1002/app.53258.

[ref8] Lei Z.; Tian D.; Liu X.; Wei J.; Rajavel K.; Zhao T.; Hu Y.; Zhu P.; Sun R.; Wong C.-P. Electrically Conductive Gradient Structure Design of Thermoplastic Polyurethane Composite Foams for Efficient Electromagnetic Interference Shielding and Ultra-Low Microwave Reflectivity. J. Chem. Eng. 2021, 424, 130365 10.1016/j.cej.2021.130365.

[ref9] Hong S.-H.; Jung D.-H.; Kim J.-H.; Lee Y.-H.; Cho S.-J.; Joo S.; Lee H.-W.; Lee K.-S.; Lee S.-Y. Electrical Conductivity Gradient Based on Heterofibrous Scaffolds for Stable Lithium-Metal Batteries. Adv. Funct. Mater. 2020, 30 (14), 1908868 10.1002/adfm.201908868.

[ref10] Sun X.; Zhao X.; Ye L. Construction of Gradient Structure in Polyetherimide/Carbon Nanotube Nanocomposite Foam and its Thermal/Mechanical Property. Compos.—A: Appl. 2019, 126, 105579 10.1016/j.compositesa.2019.105579.

[ref11] Lu W.; Li G.; Zhou Y.; Liu S.; Wang K.; Wang Q. Effect of High Hardness and Adhesion of Gradient TiAlSiN Coating on Cutting Performance of Titanium Alloy. J. Alloys Compd. 2020, 820, 153137 10.1016/j.jallcom.2019.153137.

[ref12] Wu X. L.; Jiang P.; Chen L.; Zhang F.; Yuan F. P.; Zhu Y. T. Synergetic Strengthening by Gradient Structure. Mater. Res. Lett. 2014, 2 (4), 185–191. 10.1080/21663831.2014.935821.

[ref13] Yang X.; Ma X.; Moering J.; Zhou H.; Wang W.; Gong Y.; Tao J.; Zhu Y.; Zhu X. Influence of Gradient Structure Volume Fraction on the Mechanical Properties of Pure Copper. Mater. Sci. Eng., A 2015, 645, 280–285. 10.1016/j.msea.2015.08.037.

[ref14] Jiang J.; Shen Z.; Cai X.; Qian J.; Dan Z.; Lin Y.; Liu B.; Nan C.-W.; Chen L.; Shen Y. Polymer Nanocomposites: Polymer Nanocomposites with Interpenetrating Gradient Structure Exhibiting Ultrahigh Discharge Efficiency and Energy Density (Adv. Energy Mater. 15/2019). Adv. Energy Mater. 2019, 9 (15), 1803411 10.1002/aenm.201970047.

[ref15] Wang Y.; Su D.; Ji H.; Li X.; Zhao Z.; Tang H. Gradient Structure High Emissivity MoSi2-SiO2-SiOC Coating for Thermal Protective Application. J. Alloys Compd. 2017, 703 (5), 437–447. 10.1016/j.jallcom.2017.01.317.

[ref16] Wu J.; Ju Z.; Zhang X.; Marschilok A. C.; Takeuchi K. J.; Wang H.; Takeuchi E. S.; Yu G. Gradient Design for High-Energy and High-Power Batteries. Adv. Mater. 2022, 34 (29), 2202780 10.1002/adma.202202780. 35644837

[ref17] Lu L.-L.; Lu Y.-Y.; Zhu Z.-X.; Shao J.-X.; Yao H.-B.; Wang S.; Zhang T.-W.; Ni Y.; Wang X.-X.; Yu S.-H. Extremely Fast-charging Lithium Ion Battery Enabled by Dual-gradient Structure Design. Sci. Adv. 2022, 8 (17), eabm6624 10.1126/sciadv.abm6624. 35486719 PMC9054020

[ref18] He Y.; Huang Y.; Xue R.; Shi Q.; Wu Y.; Liu R. Flexible Pressure Sensor Based on Multi-layer with Gradient Structure P(VDF-HFP)/MXene/BaTiO3 Composite Film for Human Motion Monitoring. Diam. Relat. Mater. 2023, 140, 110536 10.1016/j.diamond.2023.110536.

[ref19] Liu H.; Liu R.; Chen K.; Liu Y.; Zhao Y.; Cui X.; Tian Ye. Bioinspired Gradient Structured Soft Actuators: From Fabrication to Application. J. Chem. Eng. 2023, 461, 141966 10.1016/j.cej.2023.141966.

[ref20] Chen J.; Xu H.; Zhang C.; Wu R.; Fan S.; Zhang Y. Gradient structure enabled robust silk origami with moisture responsiveness. Chem. Eng. 2023, 454 (1), 140021 10.1016/j.cej.2022.140021.

[ref21] Ma C.; Luo H.; Liu M.; Yang H.; Liu H.; Zhang X.; Jiang L. Preparation of Intrinsic Flexible Conductive PEDOT:PSS@ionogel Composite Film and its Application for Touch Panel. J. Chem. Eng. 2021, 425, 131542 10.1016/j.cej.2021.131542.

[ref22] Gu K.; Wang S.; Li Y.; Zhao X.; Zhou Y.; Gao C. A Facile Preparation of Positively Charged Composite Nanofiltration Membrane with High Selectivity and Permeability. J. Membr. Sci. 2019, 581, 214–223. 10.1016/j.memsci.2019.03.057.

[ref23] Ozenler S.; Alkan A. A.; Gunay U. S.; Daglar O.; Durmaz H.; Yildiz U. H. Thickness Gradient in Polymer Coating by Reactive Layer-by-Layer Assembly on Solid Substrate. ACS Omega 2023, 8 (40), 37413–37420. 10.1021/acsomega.3c05445. 37841123 PMC10568690

[ref24] Yoshioka Y.; Calvert P. D.; Jabbour G. Simple Modification of Sheet Resistivity of Conducting Polymeric Anodes via Combinatorial Ink-Jet Printing Techniques. Macromol. Rapid Commun. 2005, 26 (4), 238–246. 10.1002/marc.200400527.

[ref25] De ninno A.; Gerardino A.; Girarda B.; Grenci G.; Businaro L. Top-Down Approach to Nanotechnology for Cell-on-chip Applications. Biophysics Bioengineering Lett. 2010, 3, 1.

[ref26] Zhao M.-X.; Li J.; Gao X. Gradient Coating of Polydopamine via CDR. Langmuir 2017, 33 (27), 6727–6731. 10.1021/acs.langmuir.7b01463. 28657319

[ref27] Sahu N.; Parija B.; Panigrahi S. Fundamental Understanding and Modeling of Spin Coating Process: A Review. Indian J. Phys. 2009, 83 (4), 493–502. 10.1007/s12648-009-0009-z.

[ref28] Shibata M.; Sakai Y.; Yokoyama D. Advantages and Disadvantages of Vacuum-Deposited and Spin-Coated Amorphous Organic Semiconductor Films for Organic Light-Emitting Diodes. J. Mater. Chem. C 2015, 3, 11178–11191. 10.1039/C5TC01911G.

[ref29] Tekin E.; Smith P. J.; Schubert U. S. Inkjet Printing as a Deposition and Patterning Tool for Polymers and Inorganic Particles. Soft Mater. 2008, 4, 703–713. 10.1039/b711984d. 32907172

[ref30] Singh M.; Haverinen H. M.; Dhagat P.; Jabbour G. E. Inkjet Printing—Process and Its Applications. Adv. Mater. 2010, 22, 673–685. 10.1002/adma.200901141. 20217769

[ref31] Liaros N.; Fourkas J. T. Ten years of two-color photolithography [Invited]. Opt. Mater. Express 2019, 9 (7), 3006–3020. 10.1364/OME.9.003006.

[ref32] Li J.; Zrazhevskiy P.; Gao X. Eliminating Size-Associated Diffusion Constraints for Rapid On-Surface Bioassays with Nanoparticle Probes. Small 2016, 12 (8), 1035–1043. 10.1002/smll.201503101. 26749053 PMC4815929

[ref33] Beguin A. E. Inventor. Method of coating strip material. U.S. Patent 26,816,941,954 A, 1952.

[ref34] Lin F.-H.; Liu C.-M.; Liu T.-J.; Wu P.-Y. Experimental Study on Tensioned-Web Slot Coating. Polym. Eng. Sci. 2007, 47 (6), 841–851. 10.1002/pen.20764.

[ref35] Ding X.; Liu J.; Harris T. A. L. A review of the operating limits in slot die coating processes. AIChE J. 2016, 62 (7), 2508–2524. 10.1002/aic.15268.

[ref36] Nam J.; Carvalho M. Two-Layer Tensioned-Web-Over-Slot Die Coating: Effect of Operating Conditions on Coating Window. Chem. Eng. Sci. 2010, 65 (13), 4065–4079. 10.1016/j.ces.2010.03.038.

[ref37] Chang Y.-R.; Chang H.-M.; Lin C.-F.; Liu T.-J.; Wu P.-Y. Three Minimum Wet Thickness Regions of Slot Die Coating. J. Colloid Interface Sci. 2007, 308 (1), 222–230. 10.1016/j.jcis.2006.11.054. 17234203

[ref38] Chang Y.-R.; Lin C.-F.; Liu T.-J. Start-up of Slot Die Coating. Polym. Eng. Sci. 2009, 49 (6), 1158–1167. 10.1002/pen.21360.

[ref39] Parsekian A.; Harris T. A. L. Scalable, Alternating Narrow Stripes of Polyvinyl Alcohol Support and Unmodified PEDOT:PSS with Maintained Conductivity Using a Single-Step Slot Die Coating Approach. ACS Appl. Mater. Interfaces 2020, 12 (3), 3736–3745. 10.1021/acsami.9b18936. 31880906

[ref40] Parsekian A.; Harris T. A. L. Extrusion On-demand Pattern Coating Using a Hybrid Manufacturing Process. Chem. Eng. Process. 2016, 109, 20–31. 10.1016/j.cep.2016.08.012.

[ref41] Yang Z.; Matsumoto S.; Goto H.; Matsumoto M.; Maeda R. Ultrasonic Micromixer for Microfluidic Systems. Sens. Actuators, A 2001, 93, 266–272. 10.1016/S0924-4247(01)00654-9.

[ref42] Tsai J.-H.; Lin L. Active Microfluidic Mixer and Gas Bubble Filter Driven by Thermal Bubble Micropump. Sens. Actuators, A 2002, 97–98, 665–671. 10.1016/S0924-4247(02)00031-6.

[ref43] Xu B.; Wong T.; Nguyen N.-T.; Che Z.; Chai J. Thermal Mixing of Two Miscible Fluids in a T-shaped Microchannel. Biomicrofluidics 2010, 4 (4), 044102 10.1063/1.3496359. 20981238 PMC2962670

[ref44] Ryu K. S.; Shaikh K.; Goluch E.; Fan Z.; Liu C. Micro Magnetic Stir-bar Mixer Integrated with Parylene Microfluidic Channels. Lab Chip 2004, 4 (6), 608–613. 10.1039/b403305a. 15570373

[ref45] Pamme N. Magnetism and Microfluidics. Lab Chip 2006, 6 (1), 24–38. 10.1039/B513005K. 16372066

[ref46] Yuen P.; Li G.; Bao Y.; Muller U. Microfluidic Devices for Fluidic Circulation and Mixing Improve Hybridization Signal Intensity on DNA Arrays. Lab Chip 2003, 3 (1), 46–50. 10.1039/B210274A. 15100805

[ref47] Fang J.; Li S.; Wu Z.; Chen Z. Microfluidics for Pharmaceutical Applications; Santos H. A., Liu D., Zhang H., Eds.; William Andrew Publishing, 2019; Vol. 1, pp 79–100.

[ref48] Kamholz A.; Yager P. Theoretical Analysis of Molecular Diffusion in Pressure-Driven Laminar Flow in Microfluidic Channels. Biophys. J. 2001, 80 (1), 155–160. 10.1016/S0006-3495(01)76003-1. 11159391 PMC1301222

[ref49] Holden M.; Kumar S.; Castellana E.; Beskok A.; Cremer P. Generating Fixed Concentration Arrays in a Microfluidic Device. Sens. Actuators, B 2003, 92 (1–2), 199–207. 10.1016/S0925-4005(03)00129-1.

[ref50] Jeon N.; Dertinger S.; Chiu D.; Choi I.; Stroock A.; Whitesides G. Generation of Solution and Surface Gradients Using Microfluidic Systems. Langmuir 2000, 16 (22), 8311–8316. 10.1021/la000600b.

[ref51] Irimia D.; Geba D.; Toner M. Universal Microfluidic Gradient Generator. Anal. Chem. 2006, 78 (10), 3472–3477. 10.1021/ac0518710. 16689552 PMC3770893

[ref52] Cubaud T.; Mason T. Folding of Viscous Threads in Diverging Microchannels. Phys. Rev. Lett. 2006, 96 (11), 114501 10.1103/PhysRevLett.96.114501. 16605827

[ref53] Cubaud T.; Mason T. Formation of Miscible Fluid Microstructures by Hydrodynamic Focusing in Plane Geometries. Phys. Rev. E 2008, 78 (5), 056308 10.1103/PhysRevE.78.056308. 19113217

[ref54] Mowiol: Polyvinyl Alcohol; Clariant GmbH: Sulzbach, 1999.

[ref55] Epomin Polyment; Nippon Shokubai Co.LTD.

[ref56] Homola T.; Wu Y.; Cernak M. Atmospheric Plasma Surface Activation of Poly(Ethylene Terephthalate) Film for Roll-To-Roll Application of Transparent Conductive Coating. J. Adhes. 2014, 90 (4), 296–309. 10.1080/00218464.2013.794110.

[ref57] Zhanshayeva L.; Favaron V.; Lubineau G. Macroscopic Modeling of Water Uptake Behavior of PEDOT:PSS Films. ACS Omega 2019, 4 (26), 21883–21890. 10.1021/acsomega.9b02866. 31891066 PMC6933586

[ref58] Beu T. A.; Farcaş A. Structure and Dynamics of Solvated Polyethylenimine Chains. AIP Conf. Proc. 2017, 1916, 020001 10.1063/1.5017421.

[ref59] Parsekian A.; Jeong T.-J.; Harris T. A. L. A Process Model for Slot Coating of Narrow Stripes. J. Coat. Technol. Res. 2019, 16, 1653–1661. 10.1007/s11998-019-00233-2.

[ref60] Voigt M. M.; Mackenzie R. C.; Yau C. P.; Atienzar P.; Dane J.; Keivanidis P. E.; Bradley D. D. C.; Nelson J. Gravure Printing for Three Subsequent Solar Cell Layers of Inverted Structures on Flexible Substrates. Sol. Energy Mater. Sol. Cells 2011, 95, 731–734. 10.1016/j.solmat.2010.10.013.

[ref61] Sun K.; Zhang S.; Li P.; Xia Y.; Zhang X.; Du D.; Isikgor F. H.; Ouyang J. Review on Application of PEDOTS and PEDOT:PSS in Energy Conversion and Storage Devices. J. Mater. Sci.: Mater. Electron. 2015, 26, 4438 10.1007/s10854-015-2895-5.

[ref62] Andersson P.; Forchheimer R.; Tehrani P.; Berggren M. Printable All-Organic Electrochromic Active-Matrix Displays. Adv. Funct. Mater. 2007, 17, 3074–3082. 10.1002/adfm.200601241.

[ref63] Chow T. S. Wetting of Rough Surfaces. J. Phys.: Condens.Matter 1998, 10, 445–451. 10.1088/0953-8984/10/27/001.

[ref64] Allen L. V. Emulsions and Microemulsions. The Art, Science, and Technology of Pharmaceutical Compounding, 6th ed.; American Pharmacists Association, 2020; Chapter 19.

[ref65] Galaup C.; Auriel L.; Dubs J.; Dehoux C.; Gilard V.; Poteau R.; Retailleau E.; Biasini G.; Collin F. Blue Wine, a Color Obtained with Synthetic Blue Dye Addition: Two Case Studies. Eur. Food Res. Technol. 2019, 245, 1777–1782. 10.1007/s00217-019-03295-z.

[ref66] Kohl S.; Landmark J.; Stickle D. Demonstration of Absorbance Using Digital Color Image Analysis and Colored Solutions. J. Chem. Educ. 2006, 83 (4), 644–646. 10.1021/ed083p644.

[ref67] Buck J. W.; Andrews J. H. Localized, Positive Charge Mediates Adhesion of Rhodosporidium Toruloides to Barley Leaves and Polystyrene. Appl. Environ. Microbiol. 1999, 65 (5), 2179–2183. 10.1128/AEM.65.5.2179-2183.1999. 10224017 PMC91314

[ref68] Shi H.; Liu C.; Jiang Q.; Xu J. Effective Approaches to Improve the Electrical Conductivity of PEDOT:PSS: A Review. Adv. Electron. Mater. 2015, 1 (4), 1500017 10.1002/aelm.201500017.

[ref69] Dong C.; Yuan X.; He M.; Yao K. Preparation of PVA/PEI Ultra-Fine Fibers and Their Composite Membrane with PLA by Electrospinning. J. Biomater. Sci. Polym. Ed. 2006, 17, 631–643. 10.1163/156856206777346287. 16892725

[ref70] Zhang Y.-F.; Guo M.; Zhang Y.; Tang C. Y.; Jiang C.; Dong Y.; Law W.-C.; Du F.-P. Flexible, Stretchable and Conductive PVA/PEDOT:PSS Composite Hydrogels Prepared by SIPN Strategy. Polym. Test. 2020, 81, 106213 10.1016/j.polymertesting.2019.106213.

[ref71] Liu N.; Fang G.; Wan J.; Zhou H.; Long H.; Zhao X. Electrospun PEDOT:PSS–PVA Nanofiber Based Ultrahigh-strain Sensors with Controllable Electrical Conductivity. J. Mater. Chem. 2011, 21 (47), 18962–18966. 10.1039/c1jm14491j.

[ref72] Wu X.; Jiang P.; Chen L.; Zhu Y. T. Extraordinary Strain Hardening by Gradient Structure. Heterostructured Materials; CRC Press, 2021; pp 53–72. 10.1073/pnas.1324069111 PMC4034219 24799688

[ref73] Zhao L.-H.; Wang L.; Jin Y. F.; Ren J.-W.; Wang Z.; Jia L.-C. Simultaneously Improved Thermal Conductivity and Mechanical Properties of Boron Nitride Nanosheets/Aramid Nanofiber Films by Constructing Multilayer Gradient Structure. Compos. B Eng. 2022, 229, 109454 10.1016/j.compositesb.2021.109454.

[ref74] Zhang Y.; Chi Q.; Liu L.; Zhang T.; Zhang C.; Chen Q.; Wang X.; Lei Q. PVDF-Based Dielectric Composite Films with Excellent Energy Storage Performances by Design of Nanofibers Composition Gradient Structure. ACS Appl. Energy Mater. 2018, 1, 6320–6329. 10.1021/acsaem.8b01306.

[ref75] Yang J.; Liao X.; Wang G.; Chen J.; Guo F.; Tang W.; Wang W.; Yan Z.; Li G. Gradient Structure Design of Lightweight and Flexible Silicone Rubber Nanocomposite Foam for Efficient Electromagnetic Interference Shielding. J. Chem. Eng. 2020, 390, 124589 10.1016/j.cej.2020.124589.

[ref76] Tu S.; Tian T.; Lena Oechsle A.; Yin S.; Jiang X.; Cao W.; Li N.; Scheel M. A.; Reb L. K.; Hou S.; Bandarenka A. S.; Schwartzkopf M.; Roth S. V.; Müller-Buschbaum P. Improvement of the thermoelectric properties of PEDOT:PSS films via DMSO addition and DMSO/salt post-treatment resolved from a fundamental view. J. Chem. Eng. 2022, 429, 132295 10.1016/j.cej.2021.132295.

