# Supplementary material for: Scaled Production of Functionally Gradient Thin Films Using Slot Die Coating on a Roll-to-Roll System
Source: ACS Appl Mater Interfaces. 2024 Feb 8;16(7):9264–74. doi: 10.1021/acsami.3c17558 (PMC10895578; doi:10.1021/acsami.3c17558)
Supplement: Supplementary file 1 — am3c17558_si_001.pdf [file am3c17558_si_001.pdf]

Supporting Information

Scaled Production of Functionally Graded Thin Films using Slot Die Coating on a Roll-to-Roll

Tae-Joong Jeong, Xiaoqing Yu, Tequila A. L. Harris*

Woodruff School of Mechanical Engineering, Georgia Institute of Technology, 813 Ferst Dr., Atlanta, GA, 30349, USA

Email: tjeong77@gmail.com

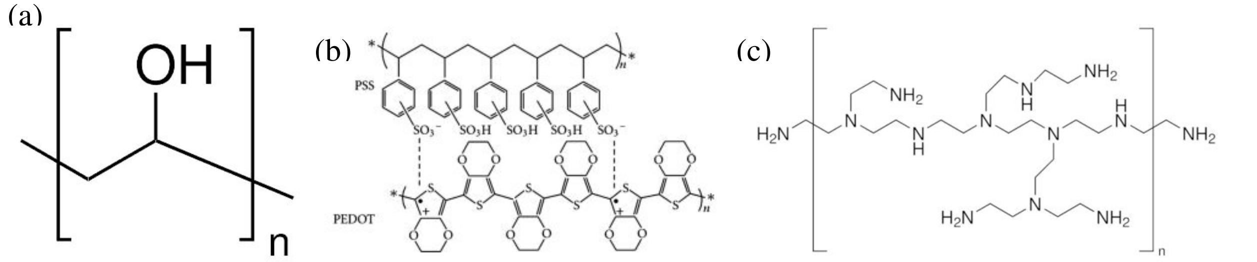

Figure S1. Chemical structures of (a) Polyvinyl alcohol (PVA)[1], (b) PEDOT:PSS[2] and (c) Polyethylenemine (PEI)[3]

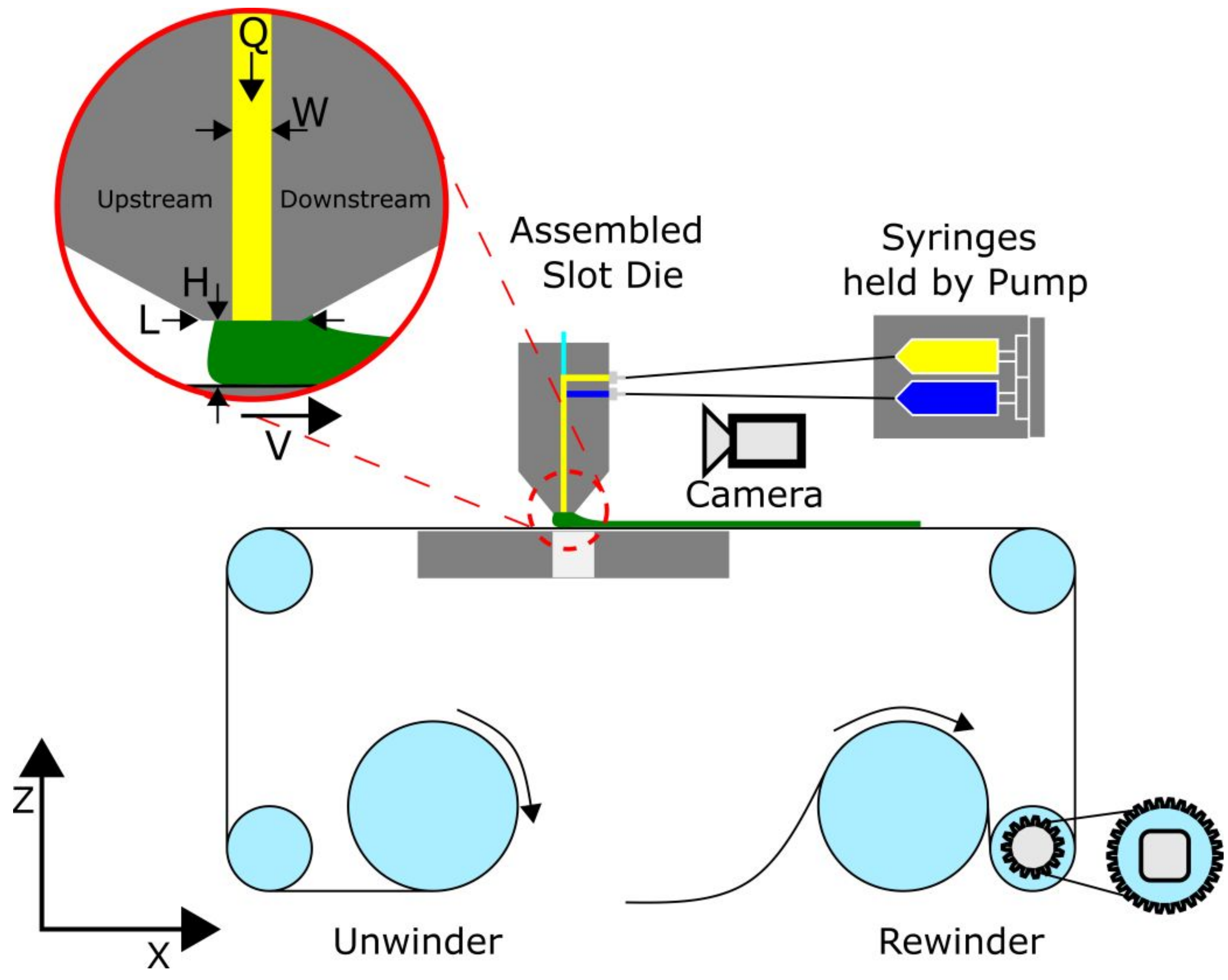

Figure S2. Schematic of overall experimental setup with important coating parameters

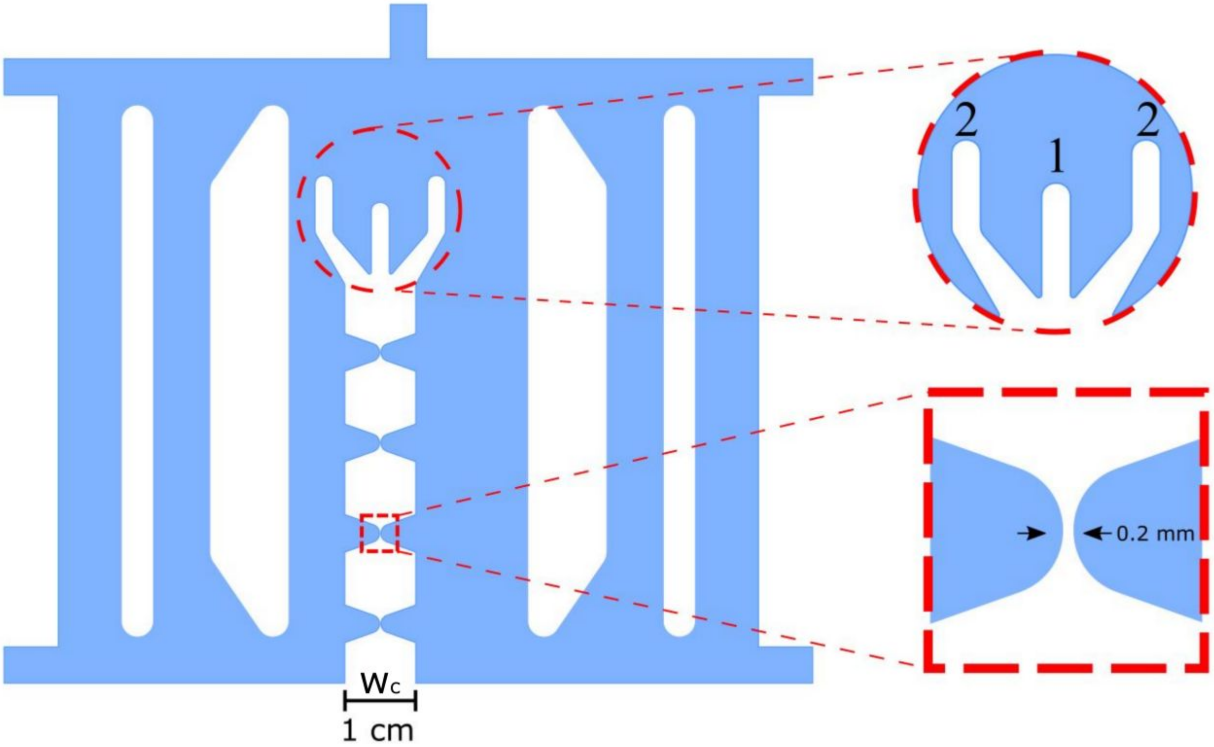

Figure S3. Schematic of shim design

[1] *Sigma-Aldrich Homepage*, https://www.sigmaaldrich.com/product/aldrich/81381

[2] Sun, K.; Zhang, S.; Li, P.; Xia, Y.; Zhang, X.; Du, D.; Isikgor, F. H.; Ouyang, J. Review on Application of PEDOTs and PEDOT:PSS in energy conversion and storage devices, *J. Mater. Sci: Mater Electron*, **2015**, *26*, 4438-4462

[3] *Sigma-Aldrich Homepage*, https://www.sigmaaldrich.com/product/aldrich/181978
